# Supplementary material for: Homokaryotic High-Quality Genome Assembly of Medicinal Fungi Wolfiporia hoelen Reveals Auto-Regulation and High-Temperature Adaption of Probable Two-Speed Genome
Source: Int J Mol Sci. 2022 Sep 10;23(18):10484. doi: 10.3390/ijms231810484 (PMC9503964; doi:10.3390/ijms231810484)
Supplement: Supplementary file 1 [file ijms-23-10484-s001.zip › ijms-1891587-Supplementary figures.pdf]

Supplementary material

Manuscript title: Homokaryotic high-quality genome assembly of medicinal fungi *Wolfiporia hoelen* reveals auto-regulation and high-temperature adaptive of probable two-speed genome

Information

1. Supplementary Figures S1 to S19

2. Supplementary Tables S1 to S16

Figure S1.

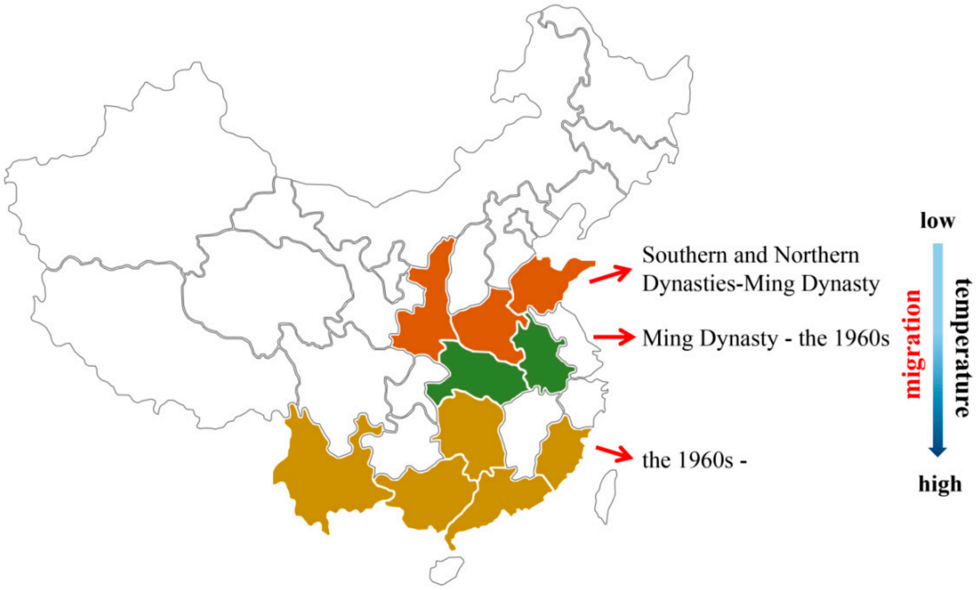

Figure S1. Migration of cultivation districts of *Wolfiporia hoelen*. The sclerotia of *Wolfiporia hoelen* were mainly cultivated in Shandong, Henan, and Shanxi Provinces in Southern and Northern Dynasties to Ming Dynasty, and in Hubei and Anhui provinces in Ming Dynasty to the 1960s, and extended to Hunan, Fujian, Guangdong, Guangxi and Yunnan Provinces in the 1960s to now.

Figure S2.

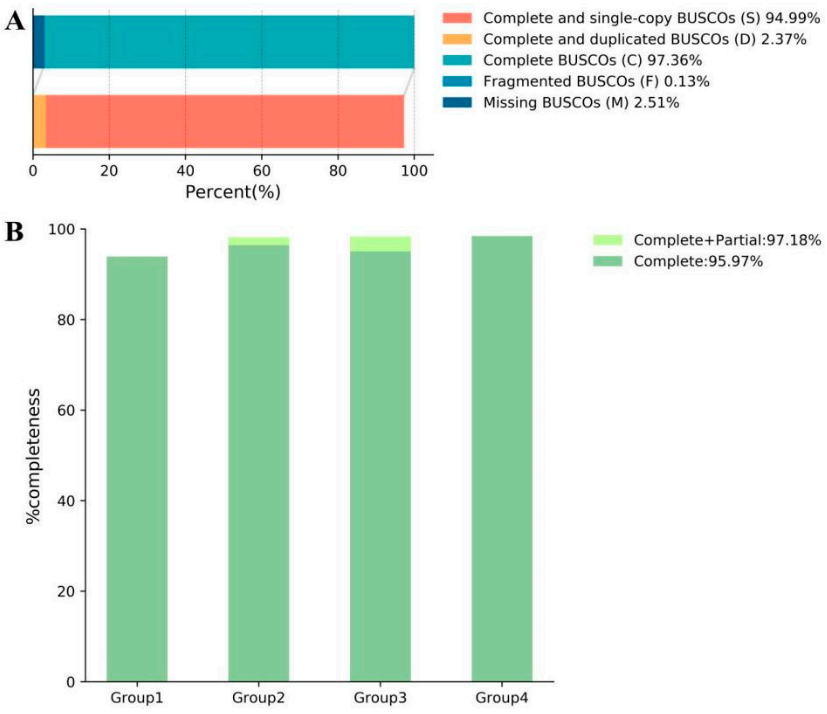

Figure S2. Genome completeness evaluation based on BUSCO and CEGMA. (A) Genome completeness evaluation based on BUSCO. (B) Genome completeness evaluation based on CEGMA.

Figure S3.

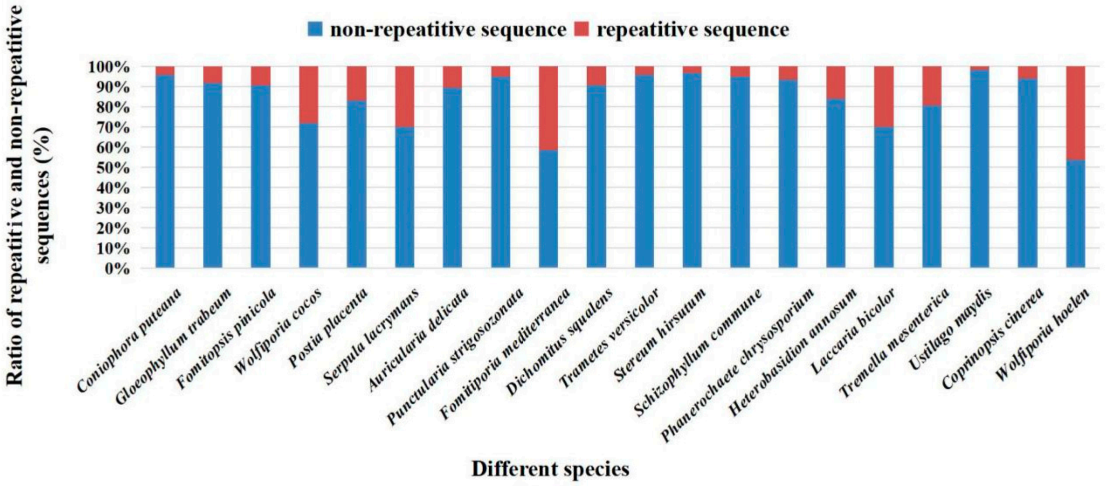

Figure S3. Repetitive sequence and non-repetitive sequence ratio of different species **Figure S4.**

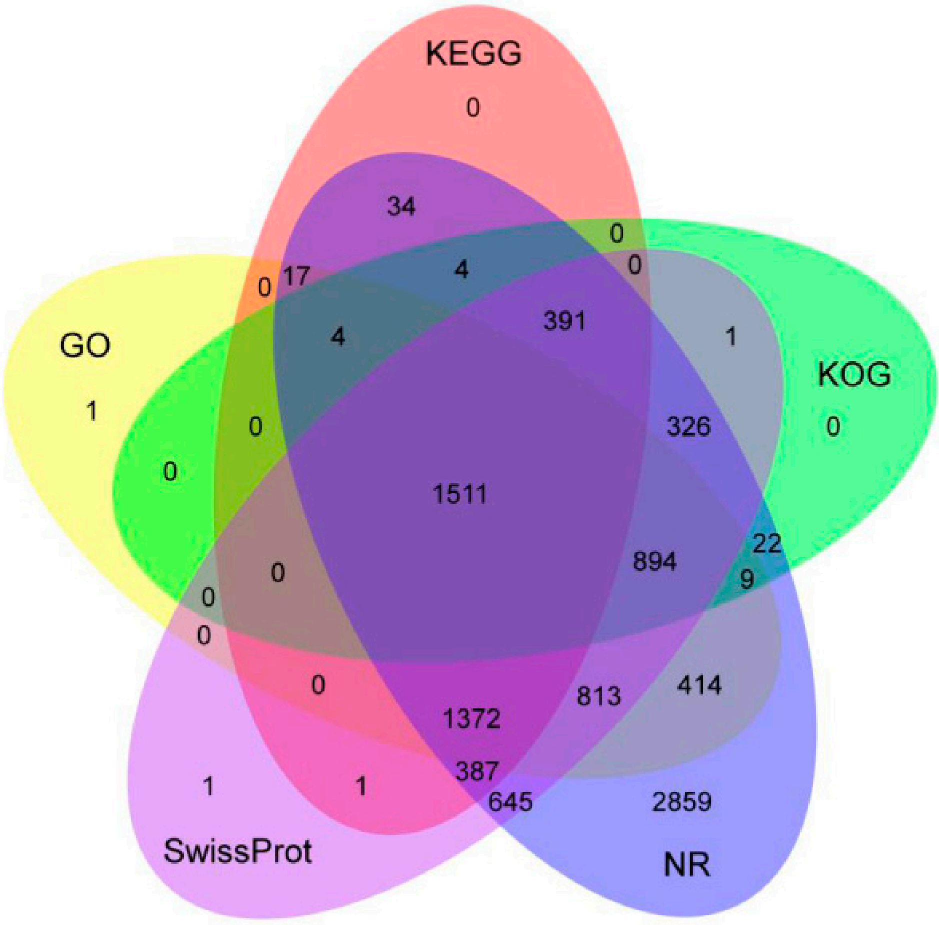

Figure S4. Venn diagram of gene annotation in different databases (NR, GO, KEGG, KOG, and SwissProt).

Figure S5.

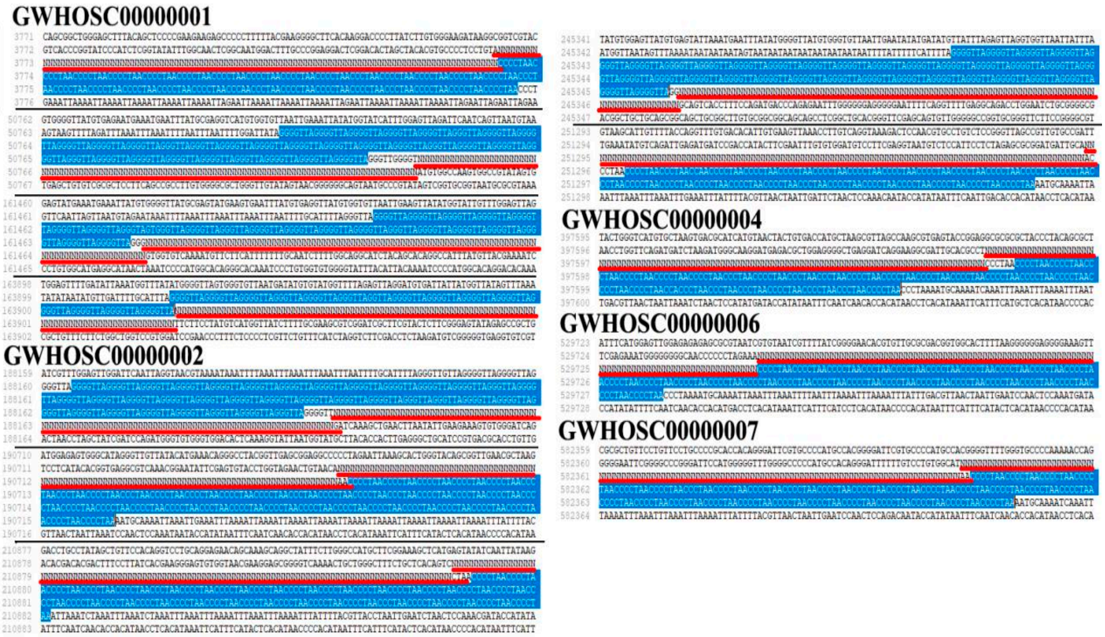

Figure S5. Telomere position on 7 chromosomes of Cao et al. [24]. Red line presented breaks connected with 100 "N", blue shade present the telomere sequences.

Figure S6.

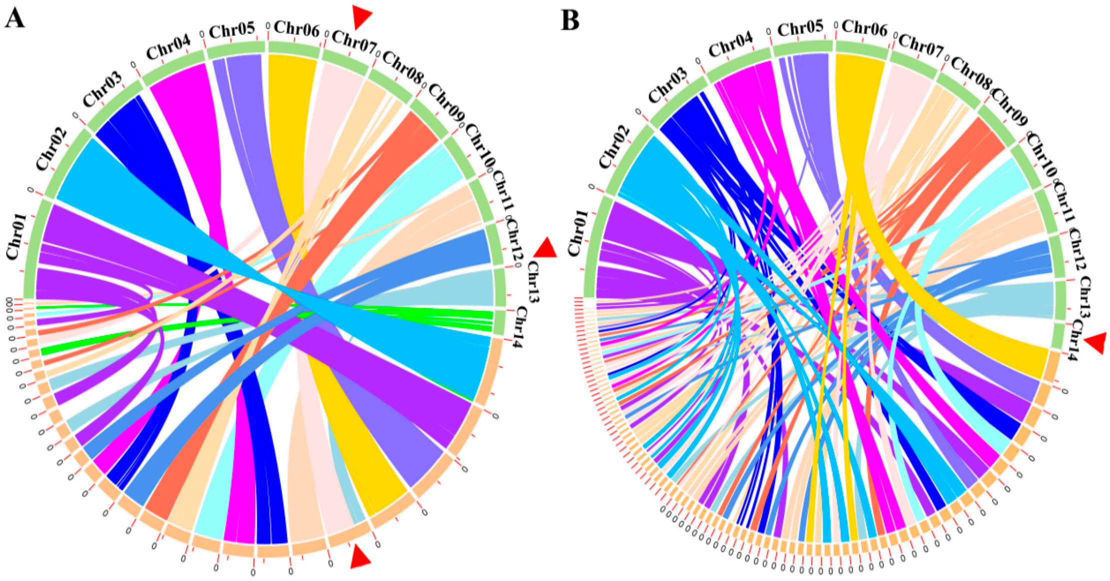

Figure S6. Whole-genome collinearity between *Wolfipoira hoelen* and *Wolfiporia cocos* and *Laetiporus sulphureus*. **(A)** Collinearity between *Wolfipoira hoelen* and *Wolfiporia cocos*; **(B)** Collinearity between *Wolfipoira hoelen* and *Laetiporus sulphureus*.

Figure S7.

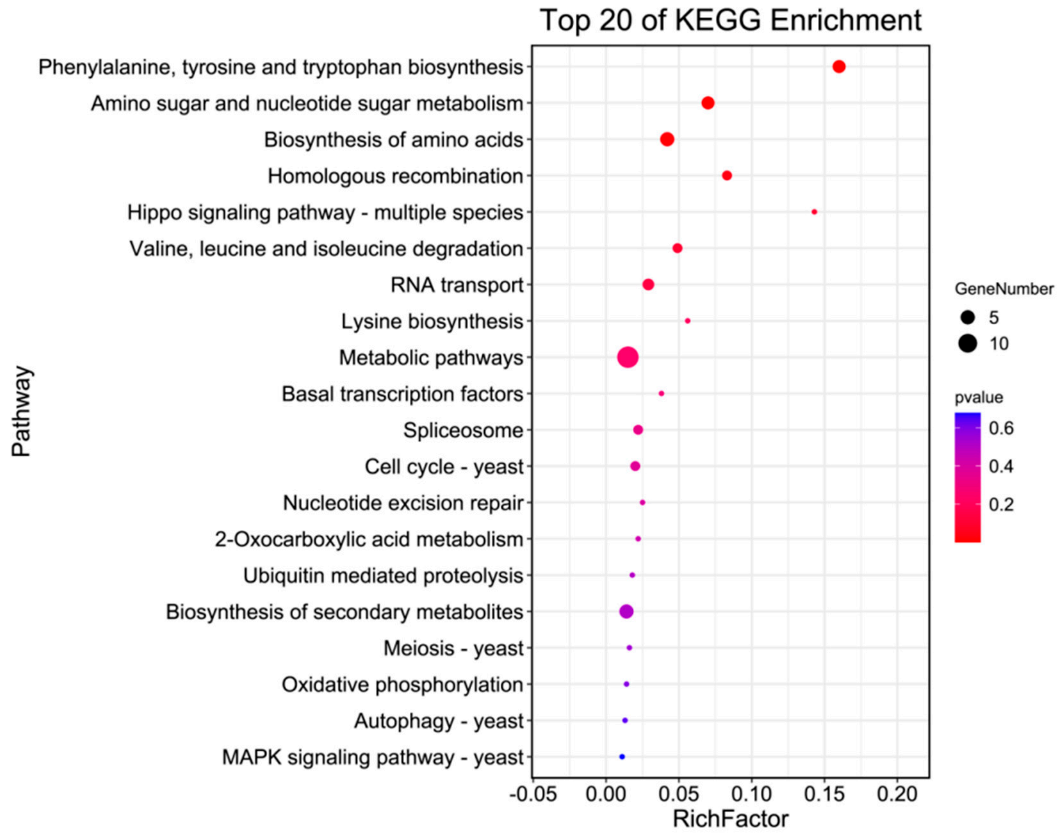

Figure S7. KEGG pathway enrichment of the genes on

Chr14. **Figure S8.**

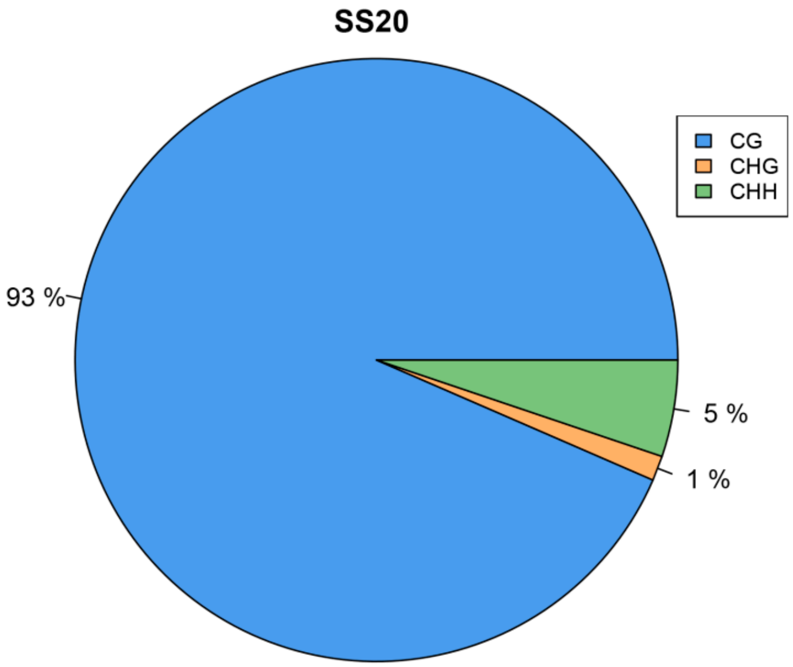

Figure S8. Ratio of different methylation types.

Figure S9.

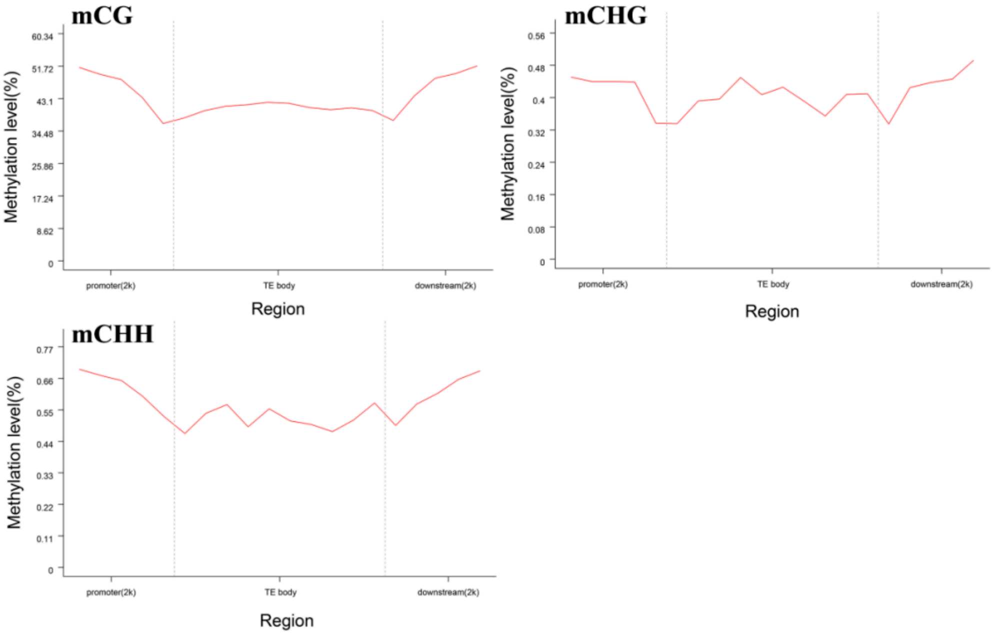

Figure S9. Level of methylation on TE body, 2kb of upstream and

downstream. **Figure S10.**

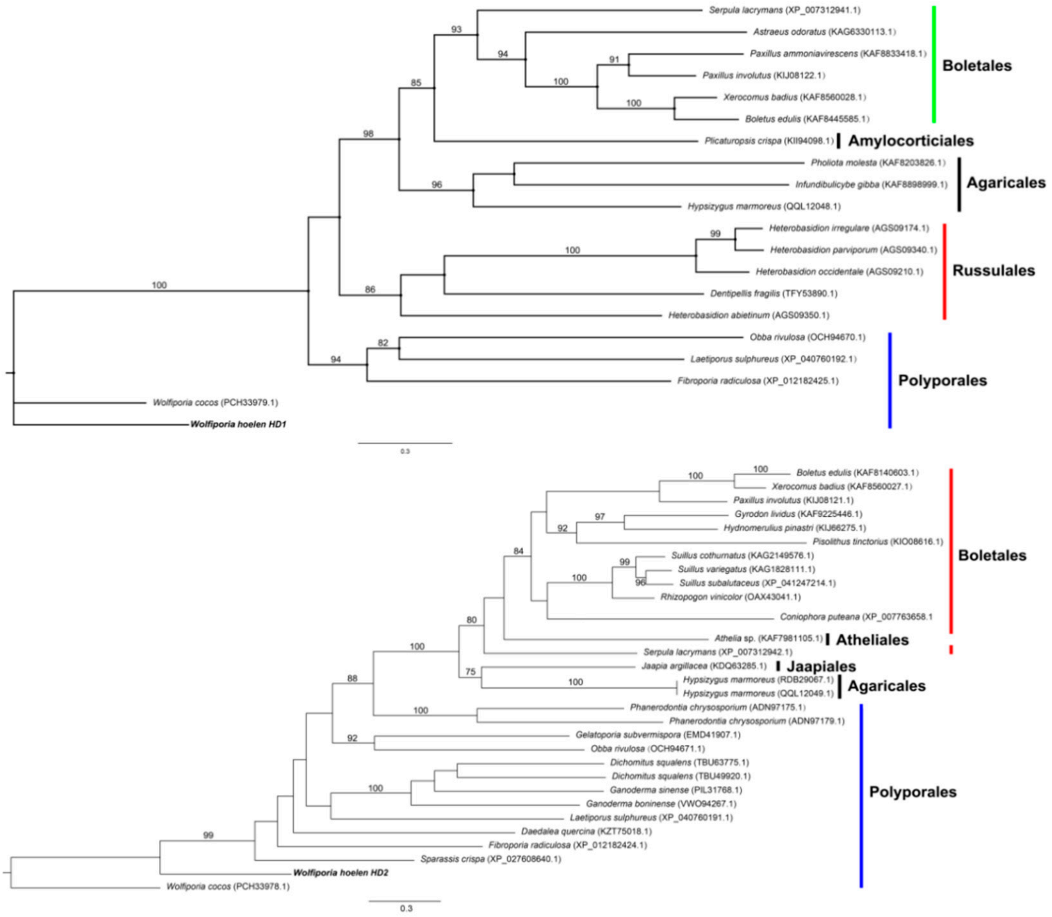

Figure S10. Phylogenetic analysis of HD1 and HD2 of *Wolfiporia hoelen* and other species inferred ML of constructed by IQ-TREE.

Figure S11.

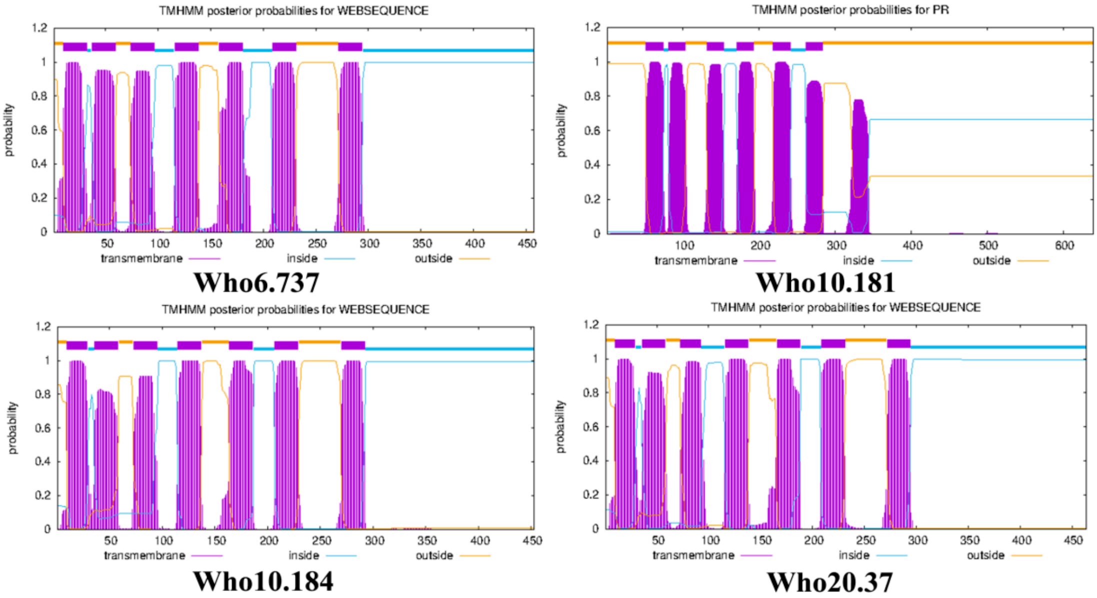

Figure S11. Transmembrane domain prediction of pheromone

receptor. **Figure S12.**

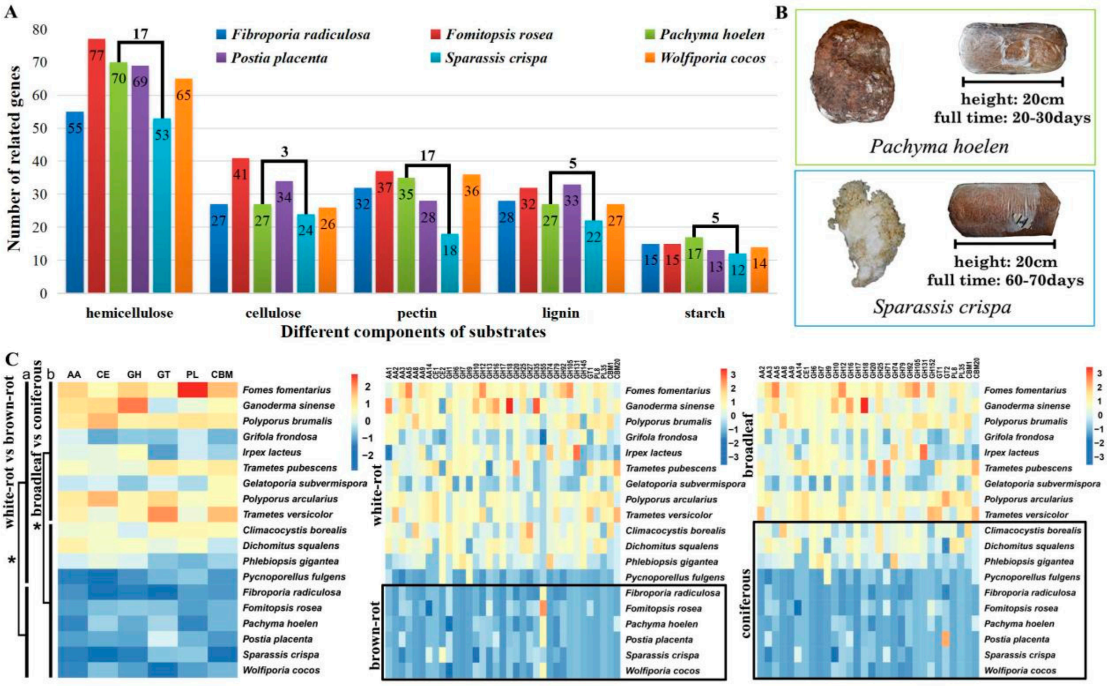

Figure S12. Comparison of CAZymes between white-rot and brown-rot, grown on coniferous and broadleaf. **(A)** Number of CAZymes related to different plant compositions between *Wolfiporia cocos* and *Wolfiporia hoelen*; **(B)** Comparison of full bag time between *Wolfiporia heolen* and *Sparassis crispa*; **(C)** Cazyme comparison between white-rot and brown-rot, grown on coniferous vs broadleaf.

Figure S13.

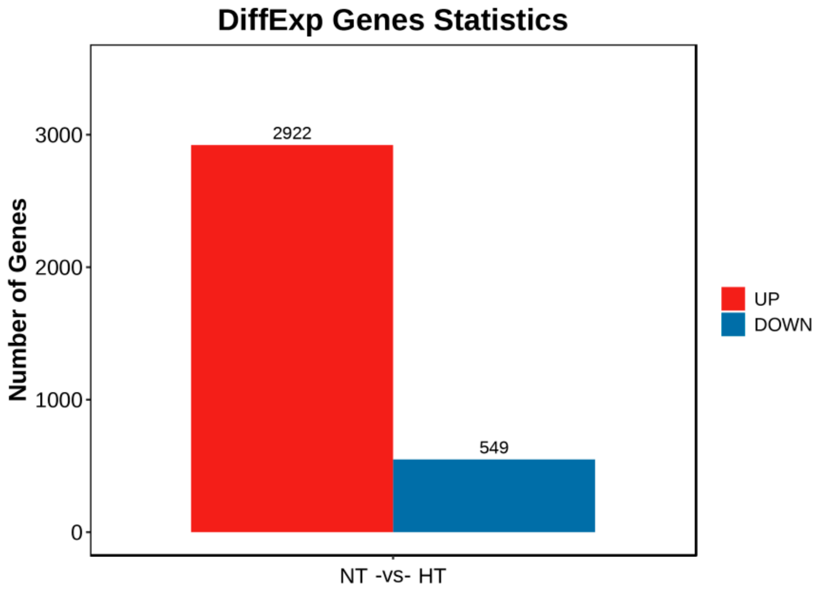

Figure S13. Number of differential expressed

genes. **Figure S14.**

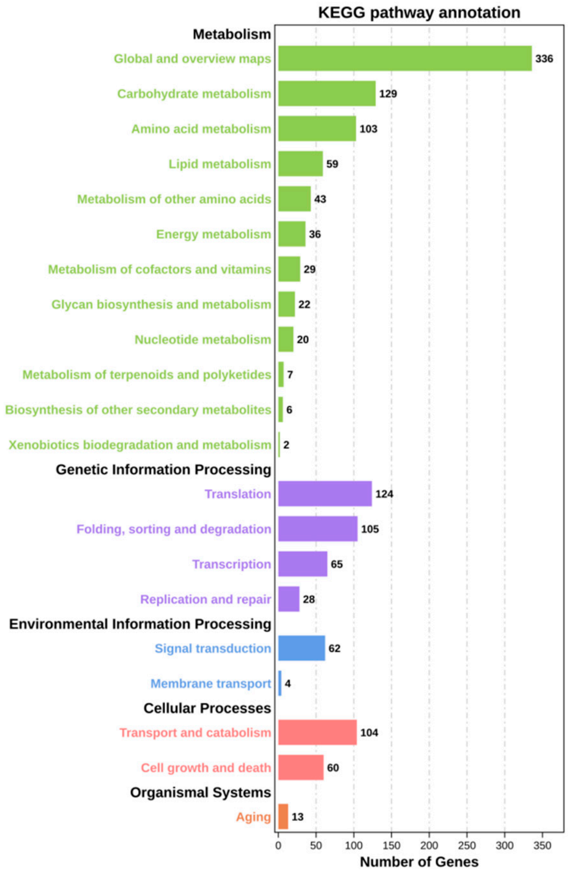

Figure S14. KEGG pathway enrichment of differential expressed genes.

Figure S15.

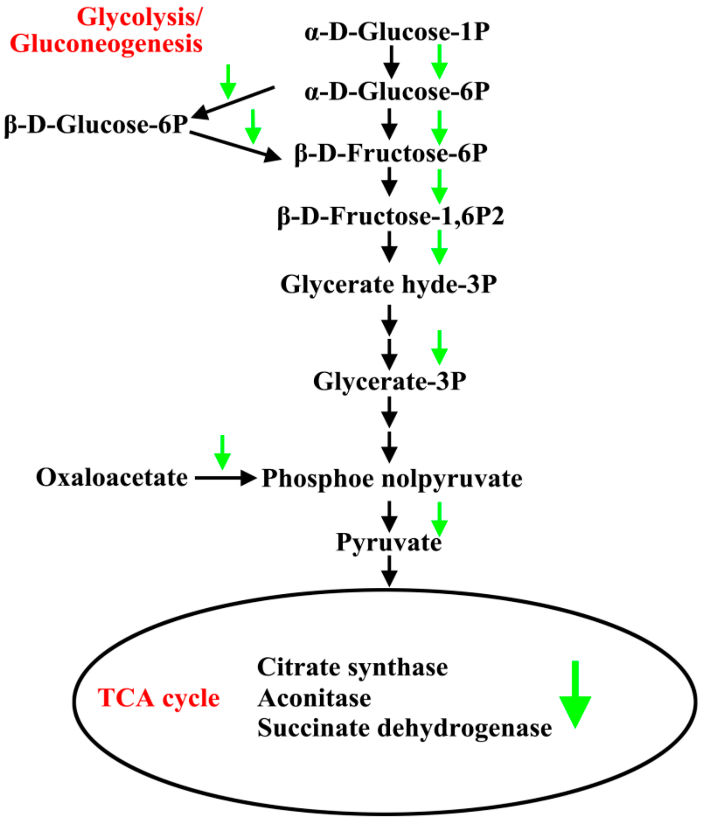

Figure S15. Down-regulated genes in Glycolysis pathway, citrate cycle pathway of HT. **Figure S16.**

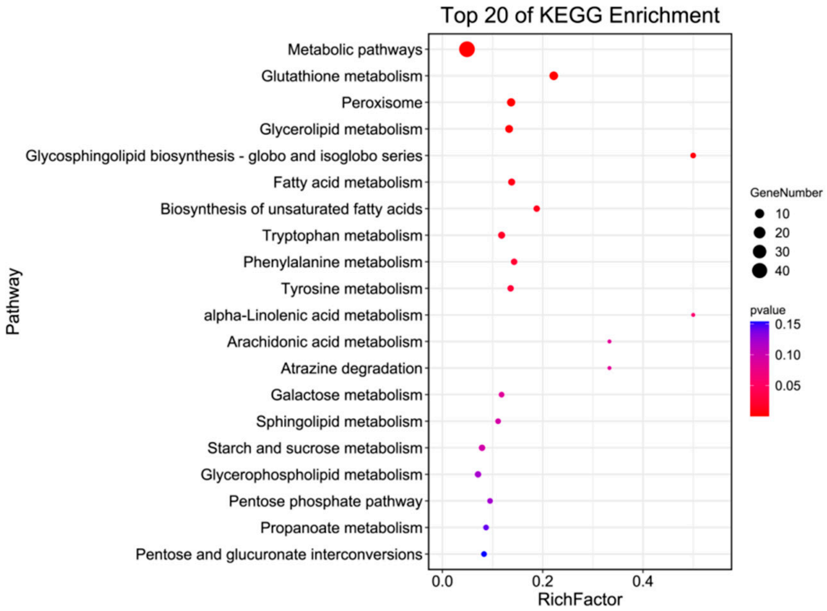

Figure S16. KEGG pathway enrichment of up-regulated genes of HT.

Figure S17.

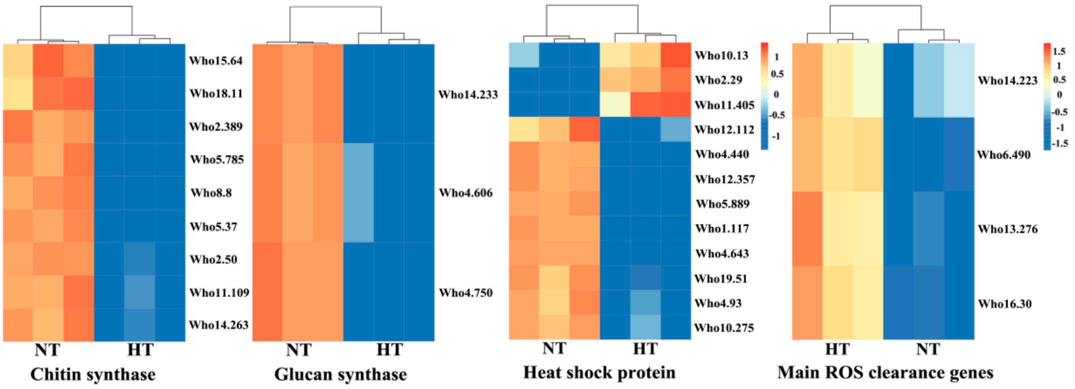

Figure S17. Up-regulated gene expression heatmap of partial function

genes. **Figure S18.**

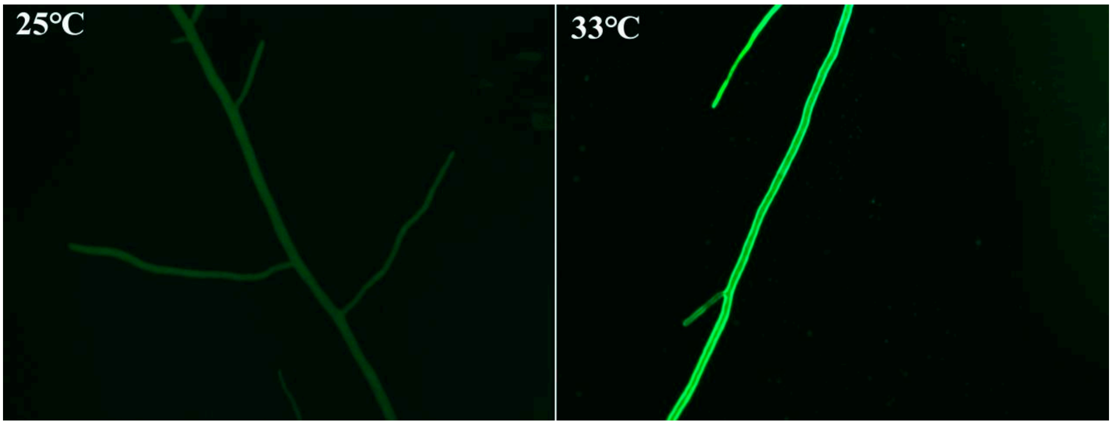

Figure S18. ROS staining of hypha cultured under 25°C and 33°C.

Figure S19.

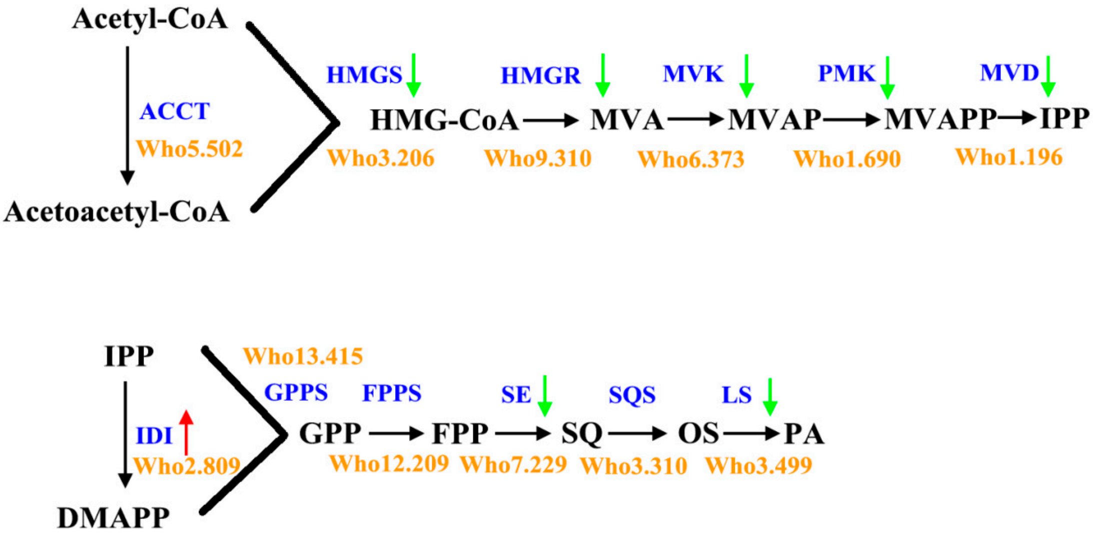

Figure S19. Differential expressed genes involved in MVA pathway for terpene biosynthetic.
